# Leprosy in elderly people and the profile of a retrospective cohort in an endemic region of the Brazilian Amazon

**DOI:** 10.1371/journal.pntd.0007709

**Published:** 2019-09-03

**Authors:** João Sérgio de Sousa Oliveira, Ana Luisa Mendes dos Reis, Luana Pereira Margalho, Geovanna Lemos Lopes, Alison Ramos da Silva, Niele Silva de Moraes, Marília Brasil Xavier

**Affiliations:** 1 Human Motion Sciences Department, Biological and Health Sciences Center, State of Pará University, Belém, Pará, Brazil; 2 Dermatoses of Sanitary Interest Research lab, Biological and Health Sciences Center, State of Pará University, Belém, Pará, Brazil; 3 Tropical Dermatology and Endemic Diseases Research Lab, Tropical Medicine Center, Federal University of Pará, Belém, Pará, Brazil; 4 Integrity Health Department, Biological and Health Sciences Center, State of Pará University, Belém, Pará, Brazil; Yale School of Public Health, UNITED STATES

## Abstract

**Background:**

Leprosy has a global presence; more than 180 thousand new cases were registered in 2013, 15% of which were found in the Americas. The elderly are a very susceptible demographic in terms of developing illnesses, mainly because of characteristics natural to the senescence of the human organism. This study’s goals were to analyze leprosy in an elderly population from a hyperendemic region of the Brazilian Amazon in a historical series from 2004 to 2013 and to determine the clinical and epidemiological profile of a series of leprosy cases of elderly people in the period spanning from 2009 to 2013.

**Methods:**

To achieve these goals, an observational, longitudinal, retrospective and descriptive study was put together to analyze leprosy in elderly people from data acquired from the Notification Aggravations Information System. Furthermore, a profile of the disease from a retrospective cohort based on data collected from medical records was developed.

**Results:**

The number of new cases and the leprosy detection rate decreased across the observed period but remained stable among the elderly. The trend for the next ten years indicates decreases in the number of cases and in the detection rate in the general population and an increase in only the elderly. The overall profile was characterized by a predominance of males (64.32%), the multibacillary clinical form (87.57%), Type 1 reaction episodes (37.50%) and some physical incapacity at diagnosis (49.19%). The risk of reaction was greater in the first six months of multidrug therapy, and the positive result from the skin smear was associated with the greater chance of reactional condition development.

**Conclusions:**

The resulting data demonstrate that leprosy amongst the elderly deserves attention because of the increased susceptibility to disability in this age group, with their higher risk of reaction and their greater level of co-morbidity.

## Author summary

Leprosy, despite being an ancient disease, still represents a challenge to public health systems today. There are still just a few studies about it, particularly among the elderly. It is known that they constitute a very heterogeneous group in terms of immune response to infections, alterations to the peripheral nervous system and predisposition to situations of vulnerability and functional dependency. The Amazon region is a hyperendemic region for leprosy and has been trying to address, along with the rest of Brazil, a rapid increase in the population’s life expectancy. This article surveys medical records from elderly people diagnosed with leprosy in a five-year period at the metropolitan region of Belém, state of Pará (Brazil), identifying a predominance of the multibacillary forms of the disease, a high prevalence of leprosy reactions mainly during treatment with multidrug therapy, and the presence of some physical incapacity in most of the people evaluated. It is expected that this study will contribute to knowledge about the clinical and epidemiological characteristics of leprosy among the elderly and stimulate the making of new studies on the theme.

## Introduction

Poverty is closely related to the occurrence of Neglected Tropical Diseases (NTDs), and leprosy has high endemicity mainly in Africa, the Americas, Southeast Asia and the Eastern Pacific, where social and economic inequalities reflect adverse conditions of life and health for the population [1]. Brazil is number two in the world for leprosy, registering more than 30 thousand new cases a year [2]. In North Brazil, the state of Pará, a region with a low Development Human Index (DHI) in the country, is considered hyperendemic with 3,917 notified cases in 2014 alone [3].

Leprosy can affect people of all ages, including the elderly. The growth of the elderly demographic is a remarkable reality. In 2000, there were already more people aged 60 and above than children aged below 15 in the world, and, according to projections, in 2050, these numbers will hold[4]. When diagnosed and treated late, leprosy leads to physical disability [5] that, when combined with the aging process and other comorbidities, can cause the loss of personal autonomy to the elderly person [6]. Additionally, leprosy reaction episodes, the phenomena potentially responsible for the functional loss of the peripheral nerves [7], also results in disability that contributes to greater vulnerability and dependency in the elderly [8].

With aging, alterations in the peripheral nervous system occur, such as a reduction in fiber myelination, decreasing nervous system conduction speed [9] and compromising the pressure and tactile senses. These alterations make the elderly person more susceptible to skin lesions that may render the leprosy diagnosis more difficult and interfere with the evaluation of these patients [10, 11].

It is necessary for health professionals, relatives and caretakers to be attentive to leprosy symptoms in elderly people, mainly in endemic areas, such that it is necessary to perform a detailed clinical investigation to make sure that diagnosis and treatment occur as early as possible. Therefore, the objective of this study was to analyze leprosy in the elderly population from the state of Pará in a historical series spanning from 2004 to 2013 and to determine the clinical and epidemiological profile of a series of leprosy cases among the elderly in a highly endemic area inside the Amazon region in the period from 2009 to 2013.

## Methods

### Ethics statement

Since this was a retrospective cohort study with elderly people, the Human Research Ethics Committee from the State University of Pará accepted to proceed to data compilation and analysis with no previous informed consent obtained from the participants (CAAE protocol number 41597615.5.0000.5174 CEP-CCBS/UEPA). With the objective of assuring the confidentiality of the collected data from the medical records, the people responsible for the health units surveyed signed a Consent Term for Database Usage. All clinical and epidemiological data were anonymized.

### Study design and settings

This study consisted of two parts, the first being an observational, longitudinal, retrospective study by means of the complete analysis of leprosy cases diagnosed in elderly people in the state of Pará, so as to observe the trend of the disease over the timeframe during which it was observed. The second part is a clinical, epidemiological approach evaluating a retrospective cohort of leprosy cases in the elderly, so as to complement the profile observed in the evaluation of the longitudinal study.

The sample of the longitudinal study was composed of new leprosy cases in the state of Pará in the historical series spanning 2004 to 2013 noted in the Notification Aggravations Information System (SINAN) and available at the Computing Department of the Unified Heath System website. These data were used to analyze leprosy trends and determine the detection rate in new cases of the disease per 10000 inhabitants among the general and elderly populations. Calculations of the detection rate were based on the values of the total resident population in the state of Pará taken from annual estimations of the Brazilian population elaborated by the Brazilian Institute of Geography and Statistics (IBGE).

The epidemiological clinical characterization was completed with data on a retrospective cohort among elderly patients diagnosed with leprosy that started and concluded treatment at the Sanitary Dermatology Specialized Reference Unit ‘Dr. Marcello Candia’, the Tropical Medicine Center at Federal University of Pará, the Basic Health Unit of Guamá and the Health School Center of Marco in the period from 2009 to 2013. Although the sample does not represent all of Pará State, we assume its importance in light of the study locations’ status as recognized leprosy centers in Pará State in addition to receiving patients from various locations and being themselves located in endemic areas. Though small, the sample proves enlightening as it relies on a multi-professional team equipped to treat leprosy, with each patient evaluated efficiently and each case handled adaptively.

### Participants

For the longitudinal study the sample was of the new leprosy cases in the state of Pará noted in the SINAN in the historical series spanning 2004 to 2013. The epidemiological clinical characterization was of the medical records in the period from 2009 to 2013. All patients 60 years-old or older were considered elderly, in accordance with the National Geriatric Policy of the Brazilian Ministry of Health.

### Variables and data sources

The types of data collected included demographics (age group and gender), clinical (operational classification, clinical form, therapeutic scheme used, disability grade at the time of diagnosis, associated comorbidities and presence of leprosy reactions) and laboratorial (smear skin index at the time of diagnosis and serology results for ELISA anti-phenolic glycolipid-1—anti-PGL-1 at the time of diagnosis). The clinical forms obeyed the Madri Classification [12] and the higher disability grade at the time of diagnosis followed the Disability Grade Classification from the World Health Organization (WHO).

The descriptions of the medical records were used to consider the reactional episodes made by a health professional and referring to clinical signs and symptoms typical to leprosy reactions or just to the type of reaction and were classified as mixed reaction (types 1 and 2) in all the patients who presented, simultaneously or not, with reaction episodes of types 1 or 2 [13]. In the absence of indication on the medical record about the type of reaction, the described reactions were classified according to the clinical criteria found in the Directives Project from the Hansenology Brazilian Society and the Dermatology Brazilian Society [14]. Patients with unclassified reactions were the ones whose medical records lacked any report of reactional signs and symptoms or classification regarding the type of reaction were registered only as “in reaction” or “in reactional state” in the medical records, such as those who presented with only clinical signs and symptoms followed by treatment without the possibility of classifying the situation because of the absence of specific characteristics of a certain type of reaction. Only reactions that occurred during the multidrug therapy and up until 24 months after medical discharge were considered, excluding reactions present at the time of diagnosis.

### Study size

For longitudinal study, a the sample was composed of 50,094 new leprosy cases in the state of Pará noted in the SINAN, corresponding to all notification in the historical series spanning 2004 to 2013. The calculation methods used for the presented detection rates were as follows? 1) Leprosy detection rate in the general population = Number of confirmed new leprosy cases in residents / Total resident population in the given period X 100,000; 2) Leprosy detection rate in children under 15 years of age = Number of confirmed new leprosy cases in residentes under 15 years of age / Total resident population in the given period X 100,000; 3) Leprosy detection rate in the elderly = Number of confirmed new leprosy cases in resident elderly people / Total resident population in the given period X 100,000. The epidemiological clinical characterization was completed with to 185 eligible medical records in the period from 2009 to 2013.

### Statistical analysis

This study analyzed the leprosy trends for the next 10 years with variables that included the detection coefficients per 10 thousand inhabitants and the number of new cases among the general population and new cases among the elderly population in a ten-year period. To obtain these values, polynomial regression models for temporal series were used with modeling of third order polynomial regression and curve adjustment models.

To analyze the data from the retrospective cohort study, measurements of central tendency (arithmetic median) and variability (standard deviation) were calculated. To verify intergroup differences, Chi-square tests or G Tests were used. Survival curves were generated using the Kaplan-Meier test to evaluate the occurrence of the first reactional episode according to the administered therapeutic scheme. The Odds Ratio (OR) was calculated between the final disclosure (leprosy reactions during and after the treatment) and the laboratorial exam results, with consideration of the 95% confidence interval (IC). The Spearman correlation non-parametric test was used to verify the degree of association between the smear skin index and the number of reactional episodes via the Pearson correlation coefficient (r).

The data were analyzed with BioEstat 5.3 software considering a significance level of 5% (p-value ≤ 0.05).

## Results

The number of new leprosy cases registered among the general population of the state of Pará in the historical series spanning the years from 2004 to 2013 was 50,094, and 5,447 of those cases included elderly individuals. There was a reduction in the number of new cases in the elderly population from 2004 to 2009 and a peak in the year 2012. There was an increasing trend between 2010 and 2012. The detection rate in the general population of the state of Pará was highly variable, diminishing from 9.61 to 4.89 per 10 thousand inhabitants. Among the elderly, the detection rate dropped throughout the years, with decreasing variation, and had a high of 0.87 and a low of 0.64 per 10 thousand inhabitants (Fig in S1 Fig).

The analysis of survival after the occurrence of leprosy reactions starting from the beginning of multidrug therapy treatment according to the operational classification and the number of doses administered to the elder individuals diagnosed with leprosy showed that the first reactional episode occurred mainly in the first six months of treatment, demonstrating that the risk for reaction was higher in the initial months of treatment and decreased progressively with time (Fig in S2 Fig).

Initially, a survey was made of 256 medical records from elderly patients diagnosed with leprosy in the aforementioned period. However, 62 patients who experienced interruptions in treatment at the surveyed units because of death, abandonment or transfer were excluded, which included 9 patients who had incomplete or inadequate medical records. The 185 remaining cases were followed from the start of treatment up until at least 24 months after the medical discharge. From the 185 elders surveyed, 64.32% were male and 69.73% were in the 60- to 69-year-old age group (67.50 years, on average). The predominant operational classification was multibacillary, and 62.70% of the elderly presented with a dimorphic clinical form. Among the therapeutic schemes used, 69.73% of the elderly went through 12 doses of multibacillary multidrug therapy (MDT/MB) and only 9.73% went through 6 doses of paucibacillary multidrug therapy (MDT/MB) (Table in S1 Table).

The occurrence of leprosy reactions in the elderly was 64.86% (Table in S2 Table). From those reactions, 37.50% presented with type 1 reactions, with a predominance of the Borderline and Lepromatous clinical forms, and 65.83% of the patients were treated with prednisone during the episodes. Among the elderly that developed reactions, only 115 presented with reactional signs and symptoms described in the medical records, and 34.78% manifested new erythematous plaques, infiltrated and/or edematous, and 21.74% manifested signs and symptoms of neuritis in type 1 reactions (Table in S3 Table).

Among the 147 elderly that went through the smear skin examination at the time of diagnosis, 65% of the patients who developed reactions presented with a positive skin examination index at the time of diagnosis. The odds ratio demonstrated that individuals with a positive skin examination index at the time of diagnosis had a 6.07 times higher chance of developing a reaction compared to individuals with negative results (p < 0.0001). The chances of developing a reaction was 2.93 times higher in those patients with a positive ELISA anti-PGL-1 serology result performed at the time of diagnosis (p = 0.1605) (Table in S4 Table).

Among the elderly, 49.19% already presented with some physical incapacity at the time of diagnosis (Table in S5 Table). The presence of disability grade 1 or 2 at the time of diagnosis was more prevalent in the Multibacillary, Borderline and Lepromatous clinical forms. Considering the comorbidities present, systemic arterial hypertension (28.65%) and diabetes mellitus (13.51%) were the most prevalent (Table in S6 Table).

## Discussion

The leprosy detection rate per 10 thousand inhabitants in the general population of the state of Pará showed a decreasing trend throughout the years. The elderly population of the state also showed a reduction in the number of cases, but the detection rate suffered a less significant drop and an approximately constant conformation. Therefore, the present study demonstrated a trend for a decrease in new cases and in the detection rate among the general population for the next ten years, and, in contrast, a trend for an increase when only the elderly population was evaluated. Such a situation among the elderly population can be explained by the higher life expectancy achieved in later decades, which resulted in a higher number of new cases diagnosed in this group.

Concerning the gender of the elders in the series of cases, there was a predominance of males that corroborates the data from Monteiro et al. [15], in which 60.3% of patients were males older than 60 years, and from Nobre et al. [16], in which there were 15.11% more males than females. However, considering that the population studied was composed of elders and that there was a feminization of the aging process because of the higher life expectancy of women [17], a predominance of females would be expected in this study. There was not a predominance of females, probably due to the long incubation period of leprosy, which can last up to seven years [11]. Maybe the men were infected previously and manifested the signs and symptoms only at an old age. It is also possible that a late diagnosis occurred, because the time between the appearance of signs and symptoms and the diagnosis can vary from a month to seventeen years [18].

The predominance of the aforementioned 60- to 69-year-old age group can be explained by the fact that it is the major age group among the elderly in the state of Pará and in Brazil, and, because these younger elders typically have more social contact, they are more susceptible to contracting leprosy. According to data from the Brazilian Institute of Geography and Statistics [19], in the last ten years, the frequency of leprosy in individuals older than 60 years old in this age group was bigger than on all the other age groups.

There was a predominance of the multibacillary forms over the paucibacillary forms of the disease, with a preponderance of the Borderline clinical form, which was in agreement with the study from Vieira et al. [20] that found a higher prevalence of the Borderline form in the leprosy profile of all ages, summing to 42.21% of the cases found.

According to Miranzi, Pereira and Nunes [21], the occurrence of multibacillary cases has a directly proportional relation to increased age. This relation could be due to the long incubation period of the disease combined with late diagnosis.

Concerning the reactional episodes, the type 1 reaction was more frequent in the evaluated patients, such as in the studies of Pinto et al. [22] and Chabra et al. [23] who studied individuals from different age groups. In this reactional type, the active participation of T lymphocytes occurs, with tissue production of Th1 cytokines (IL-2 and IFN-α) and pro-inflammatory cytokines such as the TNF-α [24]. In the elderly, in turn, there was an increase in the number of memory T lymphocytes in relation to the naive T lymphocytes due to chronic exposure to infectious agent antigens throughout life, implicating greater cytokine production and contributing to the pro-inflammatory state in the elderly [25,26], which may explain the higher occurrence of type 1 reaction in this group of individuals.

The prednisone was the most used medicine to treat leprosy reactions, especially type 1 reactions, as expected, given that corticosteroids are recognized as the drug of choice in this reaction for its suppressive effect on the inflammatory process, diminishing the INF-ɣ and TNF-α pro-inflammatory cytokines, and for their importance in the recovery of neural functions in the post-reactional period [27].

Regarding treatment of type 2 reactions, there was greater use of thalidomide associated with prednisone both in the isolated reactions and the mixed ones, a result similar to the ones found by Teixeira, Silveira and França [28] and Nazario et al. [29] in research with patients from various age groups. In Brazil, thalidomide is the drug of choice for treating type 2 reactions because of its immunosuppressive effect, allowing most patients to reach full resolution of the skin lesions within seven days [30,13]. However, moderated and aggravated type 2 reactional episodes can occur with peripheral neuritis such that the associated use of systemic corticosteroids may be necessary [31]. Effects associated with the use of corticoids in the elderly relate primarily to comorbidities that accompany aging, such as hypertension, muscular atrophy, and osteoporosis, for example. Effects related to the use of corticoids in the elderly may be diminished by the use of prescriptions only in severe episodes, such as leprous reactions, besides gradual reduction of the dosage [32, 33].

The analysis of survival based on the occurrence of leprosy reactions in relation to time demonstrated that the first reactional episode occurred mainly in the first six months, both in paucibacillary and multibacillary individuals. In the initial months of multidrug therapy treatment, the risk of occurrence was higher and diminished progressively throughout the months. In general, the reactional episodes appeared in the first six months of multidrug therapy in virtue of the rapid destruction of the bacilli by the medicine, which increases the risk of reactions considerably [34,35].

This study presented evidence of the importance of smear skin index elevation as a risk factor for the development of the reaction from the observation of a positive association between the smear skin index at the time of diagnosis and the number of reactional episodes during and after multidrug therapy. Additionally, the present study verified the higher chance of developing reactions when compared to individuals with negative results on this same test. Such findings are in agreement with the results found in the studies of Antunes et al. [36] and Brito et al. [35] and support the causal association between the bacillary load and the development of reactional states in the scientific literature.

Positive ELISA anti-PGL-1 serology at the time of diagnosis did not represent a predictive factor relevant to the occurrence of reactions during and after MDT treatment in the present research. There is a possibility, however, that the studied variables did not have a positive association due to the low number of patients who had the test, given that the serology needed to investigate leprosy is not obligatory for a diagnosis in Brazil.

In relation to the physical capacity evaluation, 95.68% of the elders were evaluated at the time of diagnosis in a way that the health units surveyed followed the recommendation of the Health Ministry, which indicate that a physical disability evaluation must be performed in at least 90% of the leprosy patients at the time of diagnosis and at the time of discharge to be considered active and of good service quality [37].

The greater prevalence of grade 1 physical incapacity among the patients with the presence of a physical disability is a piece of data that must be considered with caution because the evaluation of sensibility in the elderly can be compromised by the neurological alterations resulting from the senescence process in such a way that the altered sensibility as an outcome of leprosy can also be associated with aging itself. There may be reduction in the sense acuity in elders due to morphological alterations, size, density and location of the nociceptors in a way that, as aging progresses, more distance or touch pressure is needed for touch be perceived [38].

The more frequent comorbidities in the present study were arterial hypertension and diabetes mellitus, corroborating the data from Perry [39], whose research about the life quality of people with leprosy from all age groups found that diabetes mellitus and systemic arterial hypertension were the most frequent comorbidities among these patients and can, combined with leprosy, contribute to the installation and aggravation of physical disability and interfere in the social and economic lives of the patients.

Because the elderly constitute a population with a tendency to have health problems, in such a way that it is estimated that 80% of them suffer from at least one chronic disease, this increase in the number of chronic diseases is directly related to the higher functional incapability [40]. It would be valid that the health services that care for elders with leprosy incorporated their routine evaluation scales of functional capacity in this population. In this way, it would be possible to more completely evaluate the impact of the disease on the quality of life of these individuals, facilitating the institution of early rehabilitation.

The limitations of the present study are related to its design as it is about a retrospective cohort performed from the review of medical records, and the quality and veracity of the data entries made by the medical professionals are factors that interfere with the trustworthiness of the analyzed data. In the face of research scarcity about leprosy in the elderly, it is suggested that new prospective studies be made to contribute to greater knowledge about this theme and to the creation of strategies for early diagnosis and disability prevention, seeking to decrease costs in the health system, loss of family relationships and compromises to the autonomy of the elderly.

## Conclusion

The temporal analysis of leprosy among the elderly in the state of Pará demonstrated increasing trends for new cases and for the detection rate in the general population and a trend for an elevation in these values in the elderly population for the next ten years.

Regarding the epidemiological and clinical profile, it was verified that there was a predominance of males in the 60 to 69 year-old age group and a predominance of the multibacillary operational classification. Leprosy reactions were highly prevalent, and the first reactional episode occurred most frequently in the first six months of multidrug therapy. Patients with positive smear skin at the time of diagnosis presented higher chances of developing leprosy reactions. However, positive ELISA anti-PGL-1 serology in the diagnosis was not a predictive factor relevant to the occurrence of the reactions.

Prednisone was the most used medicine in the treatment of the reactional episodes. A high proportion of the elders already presented with some physical incapacity at the time of diagnosis. Systemic arterial hypertension and diabetes mellitus were the predominant comorbidities. Therefore, the leprosy amongst the elderly deserves attention because of the increased susceptibility to disability in this age group, with their higher risk of reaction and their greater level of co-morbidity.

## Supporting information

S1 Checklist STROBE checklist. (DOC)

S1 Fig New cases and the leprosy detection rate in the general population, children under 15 years old and elderly in the state of Para, Brazil, by year. (TIF)

S2 Fig Survival analysis of the occurrence of leprosy reactions. (TIF)

S1 Table Distribution of the elderly patients according to gender, age group, operational classification, clinical form, therapeutic scheme and number of doses used in a retrospective cohort of leprosy patients in an endemic region of the Brazilian Amazon. Source: Research Protocol, 2014.
(DOC)

S2 Table Distribution of elderly patients according to the occurrence of leprosy reactions in a retrospective cohort of leprosy patients in an endemic region of the Brazilian Amazon. Source: Research Protocol, 2014.
(DOC)

S3 Table Distribution of elderly patients according to the clinical characteristics and the type of leprosy reactions in a retrospective cohort of leprosy patients in an endemic region of the Brazilian Amazon. Source: Research Protocol, 2014. * The same patient may have presented more than one of the characteristics over the course of the clinical manifestations presented up until diagnostic discharge.
(DOC)

S4 Table Distribution of elderly patients according to the qualitative results of the bacterial index and PGL– 1 ELISA test in the diagnosis and the occurrence of leprosy reactions in a retrospective cohort of leprosy patients in an endemic region of the Brazilian Amazon. Source: Research Protocol, 2014.
(DOC)

S5 Table Distribution of elderly patients according to the disability grade at the time of diagnosis and the clinical forms in a retrospective cohort of leprosy patients in an endemic region of the Brazilian Amazon. Source: Research Protocol, 2014.
(DOC)

S6 Table Distribution of elderly patients according to comorbidities at the time of diagnosis in a retrospective cohort of leprosy patients in an endemic region of the Brazilian Amazon. Source: Research Protocol, 2014.
(DOC)
