# Supplementary figures and images for: Leprosy in elderly people and the profile of a retrospective cohort in an endemic region of the Brazilian Amazon
Source: PLoS Negl Trop Dis. 2019 Sep 3;13(9):e0007709. doi: 10.1371/journal.pntd.0007709 (PMC6743788; doi:10.1371/journal.pntd.0007709)

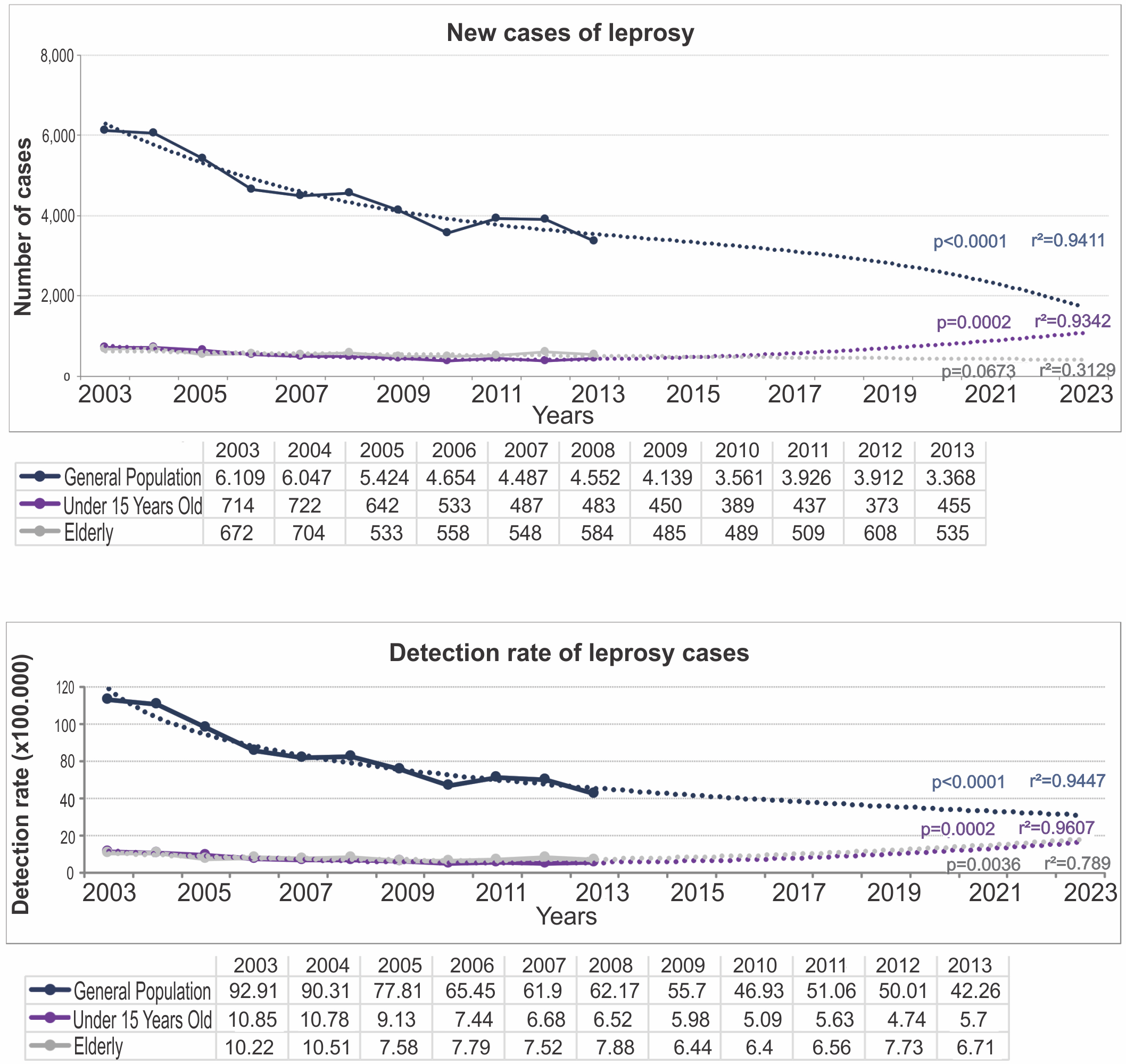

Supplement: S1 Fig — (TIF) [file pntd.0007709.s002.tif]

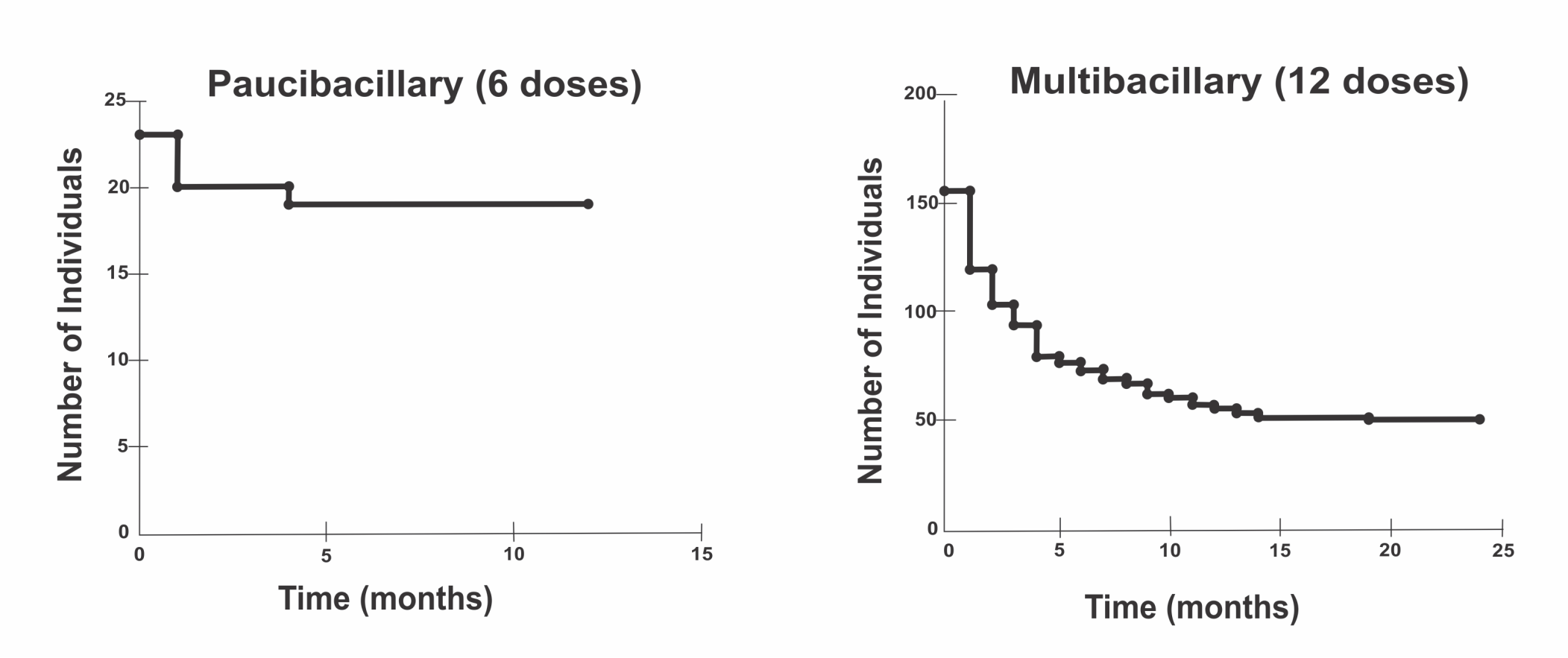

Supplement: S2 Fig — (TIF) [file pntd.0007709.s003.tif]
