# Supplementary material for: Leprosy in elderly people and the profile of a retrospective cohort in an endemic region of the Brazilian Amazon
Source: PLoS Negl Trop Dis. 2019 Sep 3;13(9):e0007709. doi: 10.1371/journal.pntd.0007709 (PMC6743788; doi:10.1371/journal.pntd.0007709)
Supplement: S1 Table — Source: Research Protocol, 2014. (DOC) [file pntd.0007709.s004.doc]

**Table 1.** Distribution of the elderly patients according to gender, age group, operational classification, clinical form, therapeutic scheme and number of doses used in a retrospective cohort of leprosy patients in an endemic region of the Brazilian Amazon.

| **General characteristics** | **N** | **%** | **Statistical Test** |
| --- | --- | --- | --- |
| **Gender** |  |  |  |
| Male | 119 | 64.32 | Chi-square  *p* < 0.0001 |
| Female | 66 | 35.68 |
| Total | 185 | 100.0 |
|  |  |  |  |
| **Age group (years)** |  |  |  |
| 60 to 69 | 129 | 69.73 | Chi-square  *p* < 0.0001 |
| 70 to 79 | 43 | 23.24 |
| 80 to 89 | 11 | 5.95 |
| 90 and above | 2 | 1.08 |
| Total | 185 | 100.0 |
|  |  |  |  |
| **Operational classification** |  |  |  |
| Paucibacillary | 23 | 12.43 | Chi-square  *p* < 0.0001 |
| Multibacillary | 162 | 87.57 |
| Total | 185 | 100.0 |
|  |  |  |  |
| **Clinical form** |  |  |  |
| Indeterminate | 4 | 2.16 | Chi-square  *p* < 0.0001 |
| Tuberculoid | 19 | 10.27 |
| Borderline | 116 | 62.70 |
| Lepromatous | 46 | 24.86 |
| Total | 185 | 100.0 |
|  |  |  |  |
| **Therapeutic scheme** |  |  |  |
| PQT/PB 6 months | 18 | 9.73 | Chi-square  *p* < 0.0001 |
| PQT/MB 12 months | 129 | 69.73 |
| Others | 38 | 20.54 |
| Total | 185 | 100.0 |
|  |  |  |  |
| **Number of doses** |  |  |  |
| 6 doses | 23 | 12.43 | Chi-square  *p* < 0.0001 |
| 12 doses | 153 | 82.70 |
| 15 doses | 2 | 1.08 |
| 24 doses | 7 | 3.78 |
| Total | 185 | 100.0 |

**Source:** Research Protocol, 2014.
