# Supplementary material for: Leprosy in elderly people and the profile of a retrospective cohort in an endemic region of the Brazilian Amazon
Source: PLoS Negl Trop Dis. 2019 Sep 3;13(9):e0007709. doi: 10.1371/journal.pntd.0007709 (PMC6743788; doi:10.1371/journal.pntd.0007709)
Supplement: S2 Table — Source: Research Protocol, 2014. (DOC) [file pntd.0007709.s005.doc]

**Table 2.** Distribution of elderly patients according to the occurrence of leprosy reactions in a retrospective cohort of leprosy patients in an endemic region of the Brazilian Amazon.

| **Leprosy reaction** | **N** | **%** | **Statistical Test** |
| --- | --- | --- | --- |
| Yes | 120 | 64.86 | Chi-square  *p* < 0.0001 |
| No | 65 | 35.14 |
| Total | 185 | 100.0 |

**Source:** Research Protocol, 2014.
