# Supplementary material for: Leprosy in elderly people and the profile of a retrospective cohort in an endemic region of the Brazilian Amazon
Source: PLoS Negl Trop Dis. 2019 Sep 3;13(9):e0007709. doi: 10.1371/journal.pntd.0007709 (PMC6743788; doi:10.1371/journal.pntd.0007709)
Supplement: S3 Table — Source: Research Protocol, 2014. * The same patient may have presented more than one of the characteristics over the course of the clinical manifestations presented up until diagnostic discharge. (DOC) [file pntd.0007709.s006.doc]

**Table 3.** Distribution of elderly patients according to the clinical characteristics and the type of leprosy reactions in a retrospective cohort of leprosy patients in an endemic region of the Brazilian Amazon.

| **Clinical characteristics** | **Type 1**  **N (%)** | **Type 2**  **N (%)** | **Types 1 and 2**  **N (%)** | **Unclassified**  **N (%)** | **Total**  **N (%)** | **Statistical Test** |
| --- | --- | --- | --- | --- | --- | --- |
| **Clinical form** |  |  |  |  |  |  |
| Indeterminate | - | - | - | - | - | G Test  *p* = 1.0000 |
| Tuberculoid | - | - | - | 4 (100.0) | 4 (3.33) |
| Borderline | 37 (47.44) | 7 (8.97) | 7 (8.97) | 27 (34.62) | 78 (65.0) |
| Lepromatous | 8 (21.05) | 13 (34.21) | 13 (34.21) | 4 (10.53) | 38 (31.67) |
| Total | 45 (37.50) | 20 (16.67) | 20 (16.67) | 35 (29.17) | 120 (100.0) |
|  |  |  |  |  |  |  |
| **Treatment** |  |  |  |  |  |  |
| Prednisone | 39 (49.37) | 6 (7.59) | 2 (2.53) | 32 (40.51) | 79 (65.83) | G Test  *p* < 0.0001 |
| Thalidomide | - | 1 (100.0) | - | - | 1 (0.83) |
| Both | - | 11 (45.83) | 13 (54.17) | - | 24 (20.0) |
| Others | 6 (37.50) | 2 (12.50) | 5 (31.25) | 3 (18.75) | 16 (13.33) |
| Total | 45 (37.50) | 20 (16.67) | 20 (16.67) | 35 (29.17) | 120 (100.0) |
|  |  |  |  |  |  |  |
| **Skin manifestations*** |  |  |  |  |  |  |
| New plaques | 40 (34.78) | 2 (1.74) | 15 (13.04) | - | - | G Test  *p* < 0.0001 |
| Oldest plaques | 5 (4.35) | - | 4 (3.48) | - | - |
| Numbness, soreness and/or accentuation of hypoesthesia in skin lesions | 6 (5.22) | - | 2 (1.74) | - | - |
| Xerosis and/or peeling | 2 (1.74) | 3 (2.61) | 3 (2.61) | 5 (4.35) | - |
| Hemorrhagic, vesiculobullous, pustular and/or ulcerative lesions | 1 (0.87) | 6 (5.22) | 4 (3.48) | - | - |
| Nodules | - | 17 (14.78) | 17 (14.78) | - | - |
|  |  |  |  |  |  |  |
| **Extracutaneous manifestations*** |  |  |  |  |  |  |
| Neural symptoms | 25 (21.74) | 10 (8.70) | 10 (8.70) | 31 (26.96) | - | G Test  *p* = 0.2558 |
| Acral edema | 14 (12.17) | 8 (6.96) | 9 (7.83) | 12 (10.43) | - |
| General ill feeling | 6 (5.22) | 9 (7.83) | 10 (8.70) | 7 (6.09) | - |
| Lymphadenomegaly | - | 1 (0.87) | 1 (0.87) | - | - |
| Rhinitis | 1 (0.87) | 1 (0.87) | - | - | - |

**Source:** Research Protocol, 2014.

*The same patient may have presented more than one of the characteristics presented during the course of the clinical manifestations until the diagnosis.
