# Supplementary material for: Leprosy in elderly people and the profile of a retrospective cohort in an endemic region of the Brazilian Amazon
Source: PLoS Negl Trop Dis. 2019 Sep 3;13(9):e0007709. doi: 10.1371/journal.pntd.0007709 (PMC6743788; doi:10.1371/journal.pntd.0007709)
Supplement: S4 Table — Source: Research Protocol, 2014. (DOC) [file pntd.0007709.s007.doc]

**Table 4.** Distribution of elderly patients according to the qualitative results of the bacterial index and PGL – 1 ELISA test in the diagnosis and the occurrence of leprosy reactions in a retrospective cohort of leprosy patients in an endemic region of the Brazilian Amazon.

| **Test Results** | **Occurrence of leprosy Reactions** | | | | **OR*** | **IC (95%)**** | ***p*-value** |
| --- | --- | --- | --- | --- | --- | --- | --- |
|  | **N** | **%** | **N** | **%** |  |  |  |
| **Bacterial Index** |  |  |  |  |  |  |  |
| Positive | 65 | 65.0 | 11 | 23.40 |  |  |  |
| Negative | 35 | 35.0 | 36 | 76.60 | 6.07 | (2.75 - 13.39) | < 0.0001 |
| Total | 100 | 100.0 | 47 | 100.0 |  |  |  |
|  |  |  |  |  |  |  |  |
| **PGL – 1 ELISA Test** |  |  |  |  |  |  |  |
| Positive | 36 | 72.0 | 7 | 46.67 |  |  |  |
| Negative | 14 | 28.0 | 8 | 53.33 | 2.93 | (0.89 - 9.63) | 0.1605 |
| Total | 50 | 100.0 | 15 | 100.0 |  |  |  |

**Source:** Research Protocol, 2014.

***** *Odds Ratio*.

******Trust interval index of 95%.
