# Supplementary material for: Leprosy in elderly people and the profile of a retrospective cohort in an endemic region of the Brazilian Amazon
Source: PLoS Negl Trop Dis. 2019 Sep 3;13(9):e0007709. doi: 10.1371/journal.pntd.0007709 (PMC6743788; doi:10.1371/journal.pntd.0007709)
Supplement: S5 Table — Source: Research Protocol, 2014. (DOC) [file pntd.0007709.s008.doc]

**Table 5.** Distribution of elderly patients according to the disability grade at the time of diagnosis and the clinical forms in a retrospective cohort of leprosy patients in an endemic region of the Brazilian Amazon.

| **Disability Grade** | | | | | | | | | | |
| --- | --- | --- | --- | --- | --- | --- | --- | --- | --- | --- |
| **Clinical Form** | **0** | | **1** | | **2** | | **Not valued** | | **Total** | **Statistical Test** |
|  | **N** | **%** | **N** | **%** | **N** | **%** | **N** | **%** | **N (%)** |  |
| Indeterminate | 4 | 100.0 | 0 | - | 0 | - | 0 | - | 4 (2.16) | G Test  *p* = 0.0920 |
| Tuberculoid | 12 | 63.16 | 4 | 21.05 | 2 | 10.53 | 1 | 5.26 | 19 (10.27) |
| Borderline | 56 | 48.28 | 38 | 32.76 | 17 | 14.66 | 5 | 4.31 | 116 (62.70) |
| Lepromatous | 14 | 30.43 | 14 | 30.43 | 16 | 34.78 | 2 | 4.35 | 46 (24.86) |
| Total | 86 | 46.49 | 56 | 30.27 | 35 | 18.92 | 8 | 4.32 | 185 (100.0) |

**Source:** Research Protocol, 2014.
