# Supplementary material for: Leprosy in elderly people and the profile of a retrospective cohort in an endemic region of the Brazilian Amazon
Source: PLoS Negl Trop Dis. 2019 Sep 3;13(9):e0007709. doi: 10.1371/journal.pntd.0007709 (PMC6743788; doi:10.1371/journal.pntd.0007709)
Supplement: S6 Table — Source: Research Protocol, 2014. (DOC) [file pntd.0007709.s009.doc]

**Table 6.** Distribution of elderly patients according to comorbidities at the time of diagnosis in a retrospective cohort of leprosy patients in an endemic region of the Brazilian Amazon.

| **Comorbidities** | **Studied group** | | **Statistical Test** |
| --- | --- | --- | --- |
|  | **N** | **%** |  |
| **Cardiovascular diseases** |  |  |  |
| Systemic Arterial Hypertension (HAS) | 53 | 28.65 | Chi-square  *p* < 0.0001 |
| Dyslipidemia | 10 | 5.41 |  |
| Cardiopathy | 7 | 3.78 |  |
| Abdominal aortic aneurysm | 1 | 0.54 |  |
|  |  |  |  |
| **Endocrine diseases** |  |  |  |
| Diabetes | 25 | 13.51 | Chi-square  *p* < 0.0001 |
| Hyperthyroidism | 1 | 0.54 |  |
|  |  |  |  |
| **Neurological diseases** |  |  |  |
| Vascular Brain Accident | 9 | 4.86 |  |
| Parkinson’s Disease | 3 | 1.62 | NA |
| Alzheimer’s Disease | 1 | 0.54 |  |
|  |  |  |  |
| **Psychiatric diseases** |  |  |  |
| Smoking | 8 | 4.32 | Chi-square  *p* = 0.2278 |
| Alcoholism | 3 | 1.62 |  |
|  |  |  |  |
| **Hepatic diseases** |  |  |  |
| Cirrhosis | 4 | 2.16 | NA |
|  |  |  |  |
| **Kidney diseases** |  |  |  |
| Chronic renal failure | 1 | 0.54 | NA |
|  |  |  |  |
| **Diseases of the respiratory tract** |  |  |  |
| Bronchial asthma | 1 | 0.54 | NA |
| Chronic bronchitis | 1 | 0.54 |  |
|  |  |  |  |
| **Osteoarticular diseases** |  |  |  |
| Arthrosis | 5 | 2.70 | NA |
| Osteoporosis | 3 | 1.62 |  |
| Herniated Disc | 1 | 0.54 |  |
|  |  |  |  |
| **Neoplastic diseases** |  |  |  |
| Prostate cancer | 1 | 0.54 | NA |
| Breast cancer | 1 | 0.54 |  |
| Lymphoma | 1 | 0.54 |  |
|  |  |  |  |
| **Infectious diseases** |  |  |  |
| Pulmonary Tuberculosis | 2 | 1.08 | NA |
| HIV | 1 | 0.54 |  |
|  |  |  |  |
| **Hematologic diseases** |  |  |  |
| G6PD deficiency | 1 | 0.54 | NA |

**Source:** Research Protocol, 2014.

NA **-** Not applicable.
